# Miticidal Tools for Management of Southern Red Mites Infesting Southern Highbush Blueberries

**DOI:** 10.3390/insects14070573

**Published:** 2023-06-21

**Authors:** Lorena Lopez, Oscar E. Liburd

**Affiliations:** 1 Entomology Laboratory, Virginia Tech’s Eastern Shore Agricultural Research and Extension Center, 33446 Research Drive, Painter, VA 23420, USA; 2 Entomology and Nematology Department, University of Florida/IFAS, 970 Natural Area Drive, Gainesville, FL 32611, USA; oeliburd@ufl.edu

**Keywords:** tetranychids, mite injury, bronzing, predatory mites, highbush blueberry, Florida

## Abstract

**Simple Summary:**

The southern red mite (SRM), *Oligonychus ilicis* is an emerging pest of southern highbush blueberries in Florida and Georgia causing significant yield and plant losses. The level of growers’ familiarity and awareness regarding this mite pest was evaluated in 2020 to evaluate miticides and contribute to miticide registrations. Growers showed confidence in identifying this pest mite but less confidence in identifying the injury caused by their feeding. Additionally, few miticide options were reported as being used by the growers, demonstrating a need for more miticidal tools and more educational material to show the availability of other effective miticides in the market. Thus, the performance of registered and non-registered miticides was assessed based on the survival of SRM and naturally occurring predatory mites. The miticides that performed the best at suppressing this pest were fenpyroximate and fenazaquin, followed by acequinocyl, bifenazate used at a high rate, and spiromesifen. Sulfur-based miticides were not effective at suppressing SRM and maintained SRM numbers comparable to the control. Demonstrating the efficacy of miticides against SRM will allow growers to diversify their pesticidal toolbox and build more diverse and effective miticide rotations that keep their blueberry plantings safe while maintaining the lifespan of the chemical products.

**Abstract:**

Tetranychid outbreaks have been detected since 2016 in southern highbush blueberries (SHB); however, it was not until 2019 that the southern red mite (SRM), *Oligonychus ilicis* (Acari: Tetranychidae) was confirmed as the pest causing severe bronzing and stunting, in multiple Florida and Georgia commercial blueberry plantings. To date, only three miticides (fenazaquin, fenpyroximate, and acequinocyl) have been registered for use in SHB and there are no clear guidelines on how to manage SRM in SHB. Similarly, there is no knowledge regarding the existence of natural enemies of SRM in SHB. This is the first report of naturally occurring predatory mites (*Amblyseius* sp. and *Neoseiulus ilicis*) associated with SRM in SHB. Predatory mites were recorded in blueberry bushes after treatment with seven miticides used to suppress SRM populations including spiromesifen, acequinocyl, sulfur, sulfur + molasses, bifenazate, fenpyroximate, and fenazaquin. The number of SRM recorded per leaf and averaged plant damage ratings (0 = no bronzing–4 = 100% bronzing) were used to evaluate miticide efficacy. Additionally, the presence or absence of predatory mites per sample was recorded. Fenpyroximate used as the standard miticide, significantly reduced mite numbers seven days after application, as well as acequinocyl and fenazaquin. Fenpyroximate and fenazaquin demonstrated the best performance for managing *O. ilicis* on SHB and treated bushes demonstrated significantly less bronzing compared with the control plants. These miticides were also safe to naturally occurring predatory mites. Lastly, the level of growers’ awareness regarding SRM was assessed using surveys in 2020 to design adequate educational materials available to the grower community.

## 1. Introduction

During the last 30 years, southern highbush blueberries (SHB) have replaced rabbiteye cultivars in Florida due to their earlier ripening and potentially high yield capacity, doubling Florida’s blueberry production capabilities [1,2]. In 2021, Florida produced 12,815 tons of berries valued at USD 78 million [3], mostly directed to the fresh market. Southern highbush blueberries are interspecific hybrids of *Vaccinium corymbosum*, *V. virgatum*, and *V. darrowi* (Ericaceae) that are well adapted to mild winter climates or “low chill” areas, such as Florida, and produce the first U.S.-produced blueberries to reach the market in early spring [2,4,5].

The blueberry bud mite, *Acalitus vaccinii* Keifer (Acari: Eriophyidae), was considered the only mite pest of blueberries that would occasionally infest SHB in Florida [6,7]. However, in 2016 a major tetranychid outbreak was reported in Florida at a commercial blueberry farm under protected structures [8]. In 2019, Florida and Georgia SHB growers experienced severe losses, estimated between USD 500,000 and USD 750,000, due to outbreaks of spider mites (Tetranychidae) [9]. The southern red mite (SRM), *Oligonychus ilicis* McGregor (Acari: Tetranychidae), was identified in 2019 as the tetranychid pest causing severe damage characterized by leaf bronzing and stunted plants in various blueberry cultivars across both states [9]. This generalist plant pest is also known as the red mite or the coffee red mite. It feeds on more than 34 host plants, most of them ornamental bushes and tree species such as camellias, azaleas, hollies, and eucalyptus. It also feeds on fruit crops such as coffee, strawberry, and cranberry [8,10].

*Oligonychus ilicis* develops several overlapping generations each year in Florida, where optimal conditions (25 ± 2 °C) can be found during the fall and spring each year. In the fall, when cool temperatures, high humidity, and dry conditions are maintained during prolonged periods of time, SRM populations increase causing economic damage; furthermore, *O. ilicis* can survive the winter without undergoing diapause [8,10,11]. In SHB, *O. ilicis* reproduce on the leaf’s lower surface, leaving a waxy and white accumulation of sheds after large populations have been established. Most SRMs are found in the mid to lower branches and start moving up the foliage as the populations grow [12]. The main symptom associated with SRM injury in ornamental and fruit crops, including SHB, is bronzed-colored leaves, as well as followed by plant stunting, and flower and fruit malformations. Additionally, the intensity of the bronzing is proportional to the degree of internal leaf damage [13].

Broad-spectrum insecticides such as pyrethroids, organophosphates, and carbamates are the main option used by growers for controlling mite and insect pests due to their effectiveness and inexpensive cost. Products like bifenthrin, abamectin, and bifenazate are frequently sprayed because are readily available on most agricultural markets and low in price. Similarly, sulfur and Sulfur-CARB seem to be used by some growers in Florida. However, farmers often spray weekly to maintain control and use a short list of chemicals in their rotation programs, turning blueberry plantings into high-chemical-input systems that can decimate natural enemies and cause secondary pest outbreaks [14,15]. Fenpyroximate is becoming a popular chemical option to suppress pest mites in Florida food cropping systems, as well as fenazaquin due to its long residual and ovicidal efficacy.

Southern red mite outbreaks have been detected in SHB for a number of consecutive years. They could become an established key pest of blueberries if integrated pest management (IPM) programs are not modified to include effective suppressive tactics against this tetranychid pest, including reduced-risk miticidal tools that are compatible with natural enemies, including predatory mites. This paper reports the evaluation of miticidal options for use in commercial SHB plantings against *O. ilicis*. Additionally, it estimates the level of awareness of Florida blueberry growers regarding SRM infestations and provides the first report of naturally occurring predatory mites associated with SRM in commercial blueberry plantings from North Central Florida.

## 2. Materials and Methods

### 2.1. Grower Survey

Data on knowledge related to SRM among blueberry stakeholders in Florida were collected to identify the level of awareness regarding SRM and design adequate educational materials available to the grower community. Data were collected during two blueberry meetings: the 2020 Florida Blueberry Growers Association (FBGA) Spring Field Day held in Citra, FL on 10 March 2020, and the 2020 Blueberry Growers Virtual Meeting on 28 July 2020. Forty blueberry growers and extension agents attended the virtual meeting, and 30 growers attended the field day. Growers participated only once in a survey that consisted of completing nine questions (10 min survey) provided by the authors of this study. The questions were set up as short open and multiple-choice questions and are listed in the appendix. The survey was responded to in person during the field day and provided as a Google Form during the virtual meeting. The questions focused on the type of blueberry plantings they grew, their confidence in identifying SRM, and their observations of mite presence or any blueberry damage symptoms caused by mite feeding. All surveys were anonymous, approved by the University of Florida’s IRB (protocol number 202000571), and consent forms were provided to the attendants prior to responding to the survey. Responses from both meetings were pooled together for analysis.

### 2.2. Plant Culture

A field trial was conducted at a commercial blueberry farm located in Waldo, FL, USA (29°47′29.4648″ N, 82°7′9.8832″ W) between 10 October and 10 November 2020. Four 152 m long rows were randomly chosen for the experiments from a three-year-old SHB planting of a proprietary cultivar (#13123) and naturally infested with SRM. Bushes were 1.5–2 m high, 1 m apart, planted in single rows (2 m apart), drip irrigated, and occasionally watered using overhead irrigation.

### 2.3. Miticide Performance

A randomized complete block design with four replicates was used to evaluate nine treatments consisting of eight miticides and water (control) (Table 1). Row sections of 12 bushes were used as experimental plots followed by five untreated bushes between plots serving as buffer zones. Additionally, one untreated row of blueberries was left between treated rows (5 m apart) as a buffer zone. Two miticide applications were conducted on 13 October and 27 October 2020 (15-day apart) using a CO_2_ sprayer with Teejet hollow cone spray cores D3 disk DC 25 (Spraying systems Co., Keystone Heights, FL, USA) and 500 L of water/ha per application. No insecticides/miticides were applied within two weeks prior to the experiments.

### 2.4. Plant Damage Assessment

An arbitrary plant damage index was used to assess the level of bronzing symptoms caused by SRM feeding on four randomly selected bushes 3-DBA (on 10 October 2020) and 14-DAA after the second application (on 27 October 2020). The index rated the percentage of bronzed foliage per plant as follows: 0 = no bronzing; 1 = 1 ≥ 25% (low bronzing); 2 = 26 ≥ 50% (moderate bronzing); 3 = 51 ≥ 75% (high bronzing); and 4 = 76 ≥ 100% (severe bronzing) bronzed foliage. Blueberries’ plant foliage was thoroughly examined for bronzing symptoms and rated by the same person at each sampling event [9].

### 2.5. Mite Collections

The mite population was assessed during seven sampling events starting three days before the first miticide application (3-DBA, pre-treatment), three, seven, and 14 days after the first and the second application (post-treatment). At each sampling event, four bushes per plot were sampled during the pre-treatment collection and tagged with colorful ribbons to avoid repeated sampling. Sampled bushes at each sampling event were tagged for differentiation. A total of 15 leaves per blueberry plant were collected in 50 mL centrifuge tubes. Leaves were washed with 10 mL of 75% ethanol per tube, shaking the tubes thoroughly for 30 s to dislodge the mites from the leaves. Leaves were discarded after, keeping only the ethanol containing the mites. Samples were checked repeatedly for adult and immature SRM under a dissecting microscope. Additionally, the presence or absence of predatory mites in the samples was recorded. A representative sample of adult pest and predatory mite specimens (~30 mites each) were slide-mounted for identification (identified by Sam Bolton, DPI, FL, USA).

### 2.6. Statistical Analysis

Spearman’s Rank Correlation Coefficients (r_s_) were calculated to identify any relationships among the survey data collected. The numbers of SRM (adults and immatures) were analyzed by fitting a generalized linear mixed model (GLMM) using GLIMMIX and following a negative binomial distribution. Plant injury data were analyzed by fitting a linear mixed model (LMM). Averaged indexes per plot were compared among treatments and sampling events (pre-treatment and 14-DAA) using the MIXED procedure. Presence and absence data for predatory mites were fitted using a GLMM with a QUAD method following a negative binomial distribution. Event “1” was equivalent to the presence of predatory mites per sample. Both GLMMs and LMMs considered the fixed effect factors of treatment, sampling event, and their interaction, together with a random effect of Block. Mean comparisons among treatments for GLMMs and LMMs were obtained by requesting LSMEANS from each procedure and the SLICE function for the effect of treatment when the GLMM was implemented. *p*-values less than 0.05 were considered significant. No transformation was used on any variable and all models and analyses were fitted using SAS 9.4 (SAS Institute, Cary, NC, USA).

## 3. Results

### 3.1. Grower Awareness of SRM

In total, 37 commercial growers participated in the survey. Responses showed that all participants grew a minimum of two SHB cultivars and up to 10 cultivars on the same farm (Table 2). Of the 26 cultivars included in the responses, more than half of the growers (57%) grew ‘Emerald’, followed by ‘Jewel’ and ‘Meadowlark’, grown by 46% and 41% of the growers, respectively. None of the growers reported growing rabbiteye cultivars. Only 27% (n = 10) of the growers reported growing other small fruits in addition to the blueberries, 5% (n = 2) reported growing fruiting vegetables, other 5% grew leafy greens, and 5% responded “other crops”.

**Figure 1:**
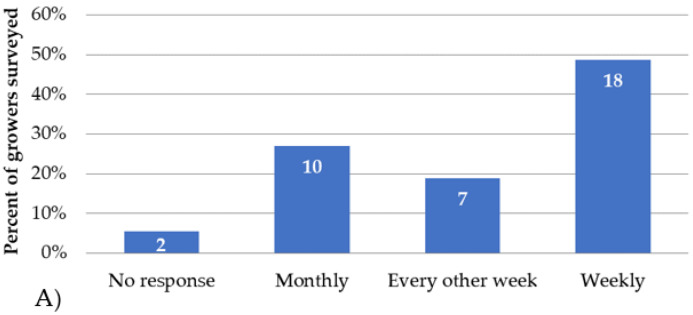

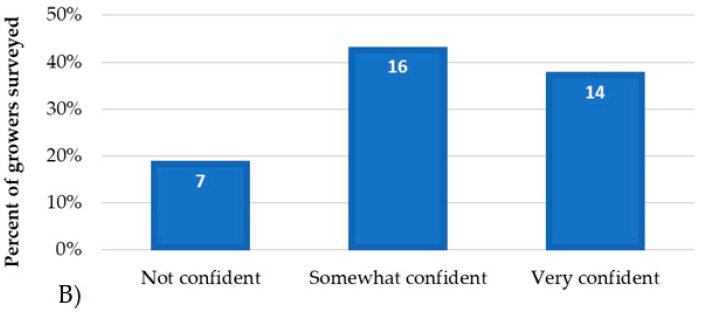
The percentage of growers and the number of growers’ responses are shown on the y-axis and within each bar, respectively, for (**A**) frequency of monitoring for pests either using traps, in situ counts, or scout services in blueberry plantings, and (**B**) level of grower confidence in identifying mite pests on their farms.

**Figure 2:**
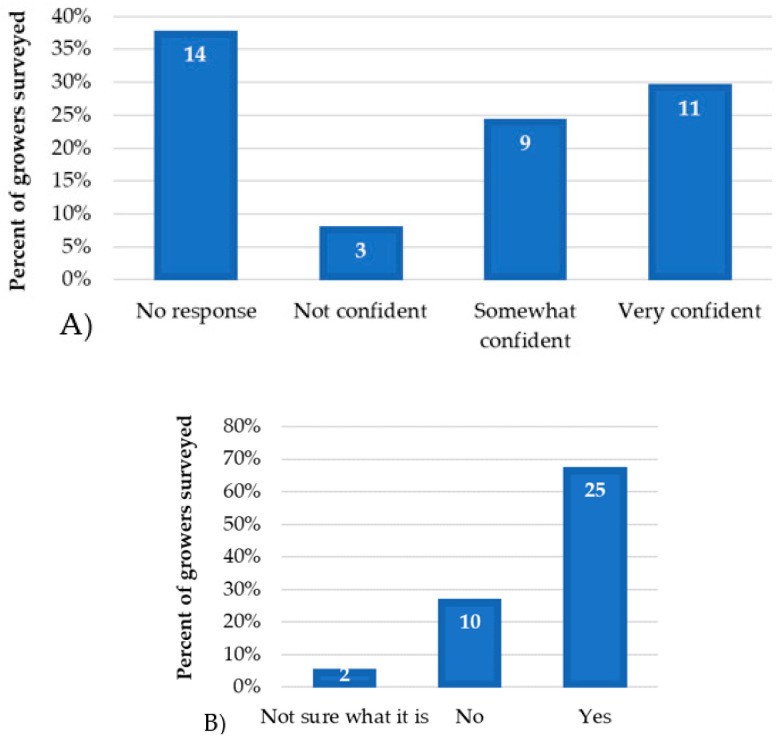
The percentage of growers and the number of growers’ responses are shown on the y-axis and within each bar, respectively, for (**A**) level of confidence in identifying mite damage and (**B**) mite damage reported by blueberry growers.

Most growers (49%) reported monitoring weekly for pests (Figure 1A), and 84% responded positively to considering mites in their monitoring practices (data not shown). However, only 38% felt very confident about their ability to identify mite pests (Figure 1B). Similarly, only 30% of the growers responded to being very confident in identifying mite damage (Figure 2A). This question (question 6 in the appendix) was only responded to during the field day held in Citra; thus, only 23 responses were collected. Due to technical difficulties, the 14 growers at the virtual meeting responded to eight questions instead of nine (shown in Figure 2A as “No response”).

Mite damage was reported as seen in commercial blueberries by 68% of the surveyed growers (Figure 2B) of which eight responded to having seen it for the first time in 2020, two since 2019, five since 2018, and eight growers in the last 3–5 years. Only 5% did not recognize the damage shown in a picture incorporated in the survey (Figure 2B).

Regarding the use of pesticides, most growers (92%, n = 34) reported using pesticides on their blueberries and responded with 20 different insecticides/miticides of which the miticide fenpyroximate (Portal) and the insecticide tolfenpyrad (Apta) were the most used (Table 3). However, most growers reported using between one and three of these pesticides, and only one reported using up to five of the 20 pesticides.

Only 49% (n = 18) reported being very confident in locating resources related to pest management and 51% (n = 19) were somewhat confident in finding these types of resources. There was a significant correlation between the confidence in mite identification and the reports of mite damage by growers (r_s_ = 0.45, *p* = 0.005, df = 36). There were no significant correlations between monitoring frequency and mite damage, number of blueberry cultivars and mite damage, confidence finding pest management resources and confidence in mite identification, monitoring frequency and pesticide use, mite damage and the use of fenpyroximate, and mite damage and the use of other miticides/insecticides (i.e., fenpyroximate, sulfur, sulfur + molasses, abamectin, bifenthrin, malathion, azadirachtin, and horticultural oils).

### 3.2. Miticide Efficacy against SRM

**Figure 3:**
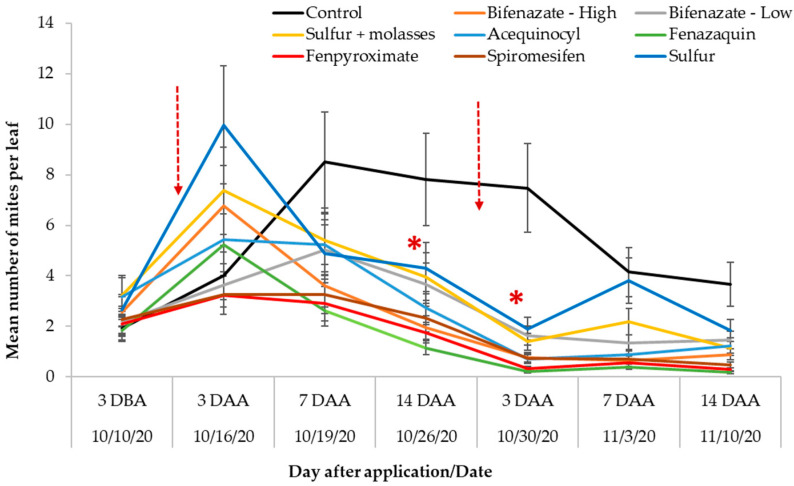
Mean (±SE) number of SRM per leaf recorded three days before miticide application (DBA), three, seven, and fourteen days after miticide applications (DAA). The dotted arrows represented two miticide applications conducted 15 days apart on 13 October 2020 and 27 October 2020. Asterisks represent significant differences for mite numbers per leaf recorded on treatments over time (Treatment * Sampling-event interaction, F_48,942_ = 3.61; *p* < 0.0001) compared with the control.

Infestation levels observed 3-DBA averaged 2.39 (±0.2) mites per leaf, with no significant differences among treatments. There was a significant treatment-by-sampling event interaction for the number of SRM per leaf (F_48,942_ = 3.61; *p* < 0.0001). The number of mites peaked three days after the first miticide application (3-DAA) in most treatments except for fenpyroximate and spiromesifen, which showed the lowest numbers, approximately two mites per leaf (Figure 3). Contrastingly, bushes treated with sulfur and sulfur + molasses showed the highest numbers of SRM compared with the rest of the treatments at 3-DAA. Most miticide treatments showed significantly fewer mites compared to the control 14-DAA and 3 days after the second miticide application. The number of mites started to decrease seven days after the first miticide application (7-DAA) across treatments, indicating a significant suppressive effect in plants treated with acequinocyl and bifenazate (high rate), as well as fenazaquin. Fenpyroximate- and spiromesin-treated plants maintained the lowest numbers of mites (one to three mites per leaf) until the end of the experiment followed by bushes treated with fenazaquin. Contrastingly, the number of mites recorded in plants treated with sulfur and sulfur + molasses showed the highest numbers among the miticide treatments over time (Figure 3).

The number of mites increased continuously in the blueberries in the control during most of the experiment, as expected. Fenpyroximate-, fenazaquin-, spiromesifen-, and bifenazate (high rate)-treated bushes showed significantly fewer mites compared to the control 7-DAA after the first miticide application. The number of SRM in all miticide treatments differed significantly from the control during the following two weeks, 14 days after the first application, and three days after the second application (Figure 3).

### 3.3. Plant Damage Caused by SRM

**Figure 4:**
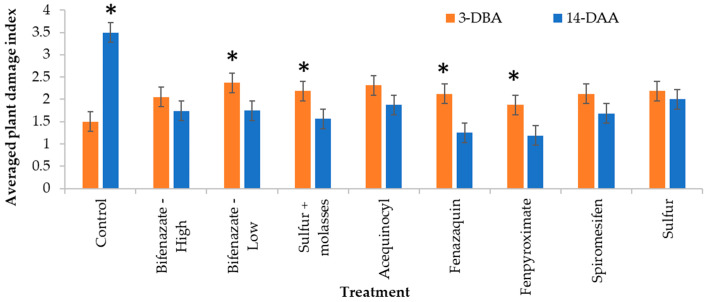
Blueberry foliage bronzed due to SRM feeding rated pre-treatment three days before the first miticide application (3-DBA, on 10 October 2020) and 14 days after the second and final miticide application on 27 October 2020 (14-DAA) based on a plant damage index (0 = no bronzing; 1 = 1 ≥ 25% (low bronzing); 2 = 26 ≥ 50% (moderate bronzing); 3 = 51 ≥ 75% (high bronzing); and 4 = 76 ≥ 100% (severe bronzing) bronzed foliage). Asterisks highlight a bar with a significantly higher average (Treatment * Sampling event interaction, F_8,267_ = 8.47; *p* < 0.0001).

Plant damage ratings recorded pre-treatment (3-DBA) were not significantly different among treatments. Moderate pre-treatment damage with an average index above two was observed in most treated plants except for plants in the control and fenpyroximate treatment with an average index below two (Figure 4).

There was a significant treatment-by-sampling event interaction (pre-treatment and 14-DAA) for the averaged plant damage index (F_8,267_ = 8.47; *p* < 0.0001, Figure 4). The percentage of bronzed foliage increased significantly in the control plants from ratings equivalent to 25% of bronzed foliage recorded at the pre-treatment up to indexes indicating high to severe (50–75%) bronzing symptoms at 14-DAA (Figure 4).

Most miticide treatments showed a significant reduction in blueberry bronzing symptoms at 14-DAA. Blueberries treated with fenazaquin and fenpyroximate showed a 0.9- and a 0.7-fold reduction in the average index, respectively, indicating a recovery from moderate bronzing closer to low bronzing symptoms (Figure 4). Plants treated with bifenazate (low rate) and sulfur + molasses also showed significant recovery symptoms on a smaller scale. There were no significant differences in bronzing symptoms recorded before and after miticide applications in the remaining treatments (Figure 4).

### 3.4. Miticide Treatment Effect on Predatory Mites

**Figure 5:**
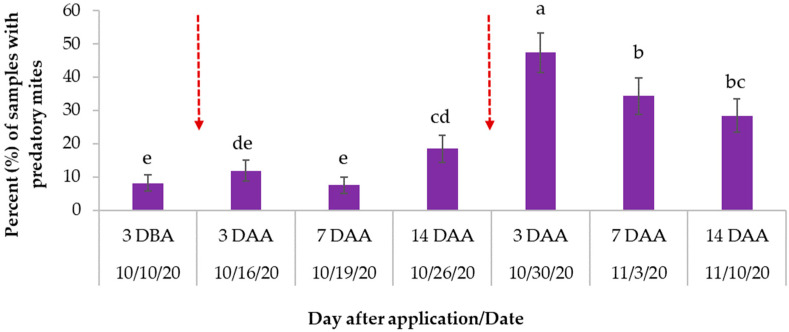
Percent (±SE) of samples with the presence of predatory mites overall treatments documented three days before miticide application (DBA), three, seven, and fourteen days after miticide applications (DAA). The dotted arrows represented two miticide applications conducted 15 days apart on 13 October 2020 and 27 October 2020. Different letters across bars indicate significant differences (sampling event main effects, F_6,990_ = 16.02; *p* < 0.0001).

Two species of predatory mites were identified in the blueberry plantings, *Neoseiulus ilicis,* a species native to Florida, and *Amblyseius* sp. (Acari: Phytoseiidae). The number of plant samples containing predatory mites differed significantly over time (F_6,990_ = 16.02; *p* < 0.0001, Figure 5). The highest numbers were observed three days after the second miticide application, with approximately 50% of the samples showing predators, indicating that predatory mites were able to survive or recolonize the plants after the miticide applications. We continued to find them in 40% and 30% of the samples in the following two weeks, respectively (Figure 5).

**Figure 6:**
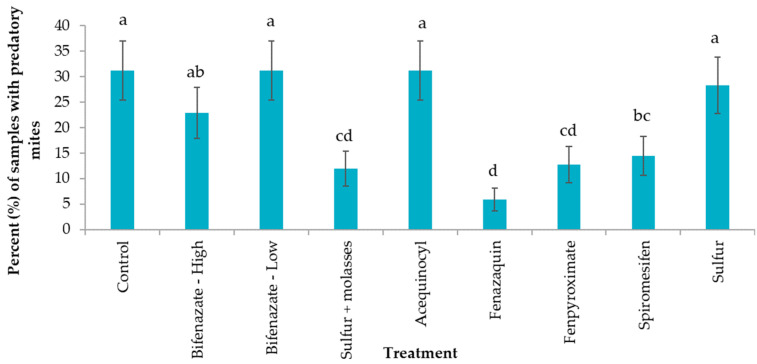
Percent (±SE) of samples with the presence of predatory mites recorded three days before miticide application (DBA), three, seven, and fourteen days after miticide applications (DAA). The dotted arrows represented two miticide applications conducted 15 days apart on 13 October 2020 and 27 October 2020. Different letters across bars indicate significant differences (treatment main effects, F_8,990_ = 6.23; *p* < 0.0001).

The percent of plant samples including predatory mites differed significantly among treatments (F_8,990_ = 6.23; *p* < 0.0001). As expected, a good percentage of samples with predatory mites were collected from the control (up to 37% of samples), followed by samples collected from bifenazate- (low rate), acequinocyl-, and sulfur-treated plants. Contrastingly, plants treated with fenazaquin showed the lowest number of samples with predatory mites, followed by fenpyroximate and sulfur + molasses treatments (Figure 6).

## 4. Discussion

Assessments for estimating blueberry growers’ level of awareness regarding SRM infestations and damage symptoms are vital to assist blueberry stakeholders and the grower community with pest management decisions, tools, and educational materials. Therefore, we designed a survey to better understand the pest management practices used by commercial blueberry growers in Florida. The survey demonstrated high levels of awareness regarding the presence of this emerging mite pest in Florida’s blueberry plantings, and most growers stated they considered mites in their monitoring practices. Similarly, the survey highlighted that growers feel confident in identifying mites, but less confident in recognizing the bronzing symptoms caused by mite feeding. Despite this, we were able to confirm that growers with higher confidence in identifying mite pests were also the growers that had a significantly higher ability to identify and report mite damage in their blueberry plantings, as expected (r_s_ = 0.454, *p* = 0.004). Additionally, some growers detected bronzing symptoms caused by SRMs as far back as 2015; however, started noticing severe symptoms in 2019 and 2020. These responses are consistent with previous reports received at the Small Fruit and Vegetable IPM Lab regarding severe mite infestations in North-Central Florida and Georgia blueberry plantings with up to 40 ha severely bronzed and stunted [9].

One of the integrated pest management (IPM) tactics that are recommended to implement in any IPM program is to schedule pesticide applications based on economic thresholds (if any) or infestation data collected during monitoring events [16,17,18]. Nonetheless, many fruit and vegetable growers prefer to schedule prophylactic or weekly insecticide/miticide sprays to protect their plantings. In the case of blueberries in Florida, insecticides are applied to 84% of the planted hectares in the state [5,16]. This is particularly common in blueberry plantings after the emergence of SWD. This seems to be common practice for most blueberry growers surveyed, since the frequency of monitoring for pests (i.e., weekly, every other week, and monthly) was not significantly related to the growers’ responses to the use of pesticides. However, the use of miticides on a weekly schedule without knowledge of the levels of infestation can cause rapid miticide resistance development and over time contribute to the establishment of SRM as a key pest in SHB [16].

The most commonly grown SHB cultivars identified in the survey were ‘Emerald’ and ‘Jewel’. These cultivars are considered the backbone of the Florida blueberry industry [15,19]. These high-yielding cultivars should be frequently monitored for SRM presence and bronzing symptoms. Because the feeding damage caused by SRM can directly affect photosynthesis, it can also indirectly affect flowering and yield if infestations are left unchecked [10,20]. The bronzing symptoms may affect the early production of berries; however, cultivars that leaf well, such as ‘Jewel’ may have the potential to recover from SRM damage. To the best of our knowledge, there have been no studies indicating any cultivar susceptibility to SRM infestations and the grower survey demonstrated that the diversity of blueberry cultivars in the same farm was not significantly related to the mite damage encountered by the growers surveyed. However, SRM has been detected in blueberry leaf samples of ‘Farthing’, ‘Avanti’, ‘Arcadia’, ‘Meadowlark’, and ‘KeyCrisp’, sent to our laboratory facilities in Gainesville, FL in 2019 and 2020, and varietal preference may be identified in future studies [12]. *Oligonychus ilicis* can cause economic damage in blueberry production if infestations are not detected and suppressed early in the season [20]. Despite causing indirect damage by feeding on the foliage of its hosts, large populations can significantly reduce photosynthesis (>50% reduction in coffee plantings), resulting in stunted plants with roughened shoots and low potential to produce flower buds [8,10,20].

The infestation patterns of SRM in blueberry plantings in Florida and Georgia follow the pattern of secondary pests’ outbreaks and we believe broad-spectrum insecticides used against key pests such as spotted-wing drosophila (*D. suzukii*, SWD), chilli thrips (*S. dorsalis*), and flea beetles (*Colaspis pseudofavosa* Riley, Coleoptera: Chrysomelidae) may be the primary driver for this phenomenon. For instance, SRM has been reported in various blueberry plantings since 2015 but was not reported in the literature until 2020, approximately seven years after SWD became a problem in Florida [14]. We hypothesize that SRM moved from one of their primary hosts grown in the southeast (ornamental plants such as boxwood, camellias, or hollies) to blueberries because their natural enemies in blueberry planting were destroyed due to the overuse of broad-spectrum insecticides. Pyrethroids are heavily used in SHB during harvest for control of SWD and post-harvest for the control of the blueberry leaf beetle (*C. pseudofavosa*) and chilli thrips (*S. dorsalis*) [3,21,22]. The non-target effects of these broad-spectrum insecticides reduce the populations of natural enemies that keep secondary pests such as SRM from increasing in numbers by killing them or limiting recolonization from natural enemies that escaped from sprays [20,22]. The removal of competitors creates an opportunity for secondary pests such as SRM to infest blueberry plantings, which can be exacerbated by the ability of *O. ilicis* to increase in abundance after exposure to low concentrations of pyrethroids in the field. This phenomenon is known as hormesis, or the stimulatory effect associated with low doses of insecticides or miticides [23]. For instance, pyrethroids such as bifenthrin are commonly known for triggering this physiological phenomenon in many spider mite populations infesting fruit crops, and it is particularly concerning that bifenthrin is reported as one of the top insecticides used by Florida blueberry growers [3]. Currently, infestations with SRM are considered outbreaks (i.e., short-term consequences of pesticide use); however, SRM could soon become a new key established pest in SHB if blueberry pest management programs do not include alternative miticide options and alternative mite management tactics.

In this study, fenpyroximate showed the best performance for control of SRM in the field trials, and it was also the only true miticide (i.e., killing only mites, and not insects) reportedly used by most growers in the survey. This miticide was effective against this pest in our 2019 trials [9] and was registered for use in SHB in 2020. Fenpyroximate has become a popular and effective tool against mite infestations in many vegetable and fruit cropping systems, and this was confirmed during the grower survey. Despite that, it is always recommended that growers have a variety of insecticidal and miticidal tools to rotate as part of their resistance management plans. However, the diversity of miticides or miticides/insecticides that growers have in their toolboxes was low based on the growers’ survey responses.

Acequinocyl and fenazaquin were also effective at reducing SRM populations in our trials in 2019 and 2020 after the second application. It is important to highlight that only one application of fenazaquin per year is permitted in blueberries. Thus, the recommended rate was split in half to conduct two applications during the trials. This modification in label rate was carried out only for experimental purposes and is not recommended for commercial use. The reduction in SRM after fenazaquin and acequinocyl applications at 14-DAA may be explained by their residual effect, which lasts for three to four weeks after application. Bushes treated with fenpyroximate and fenazaquin showed signs of recovery at the end of the experiment with a reduction in bronzing symptoms, but this was not observed in bushes treated with acequinocyl. Recent studies have reported the development of cross-resistance to acequinocyl in some populations of other species of spider mites, such as *T. urticae*. It is possible that other tetranychid species such as the SRM may be resistant to this miticide, if exposed while infesting other host plants [24,25].

Bifenazate (high rate) demonstrated potential to suppress SRM consistent with other evaluations performed by Disi et al. [26]; however, it is not registered for use in SHB yet. Similarly, spiromesifen demonstrated a good efficacy against SRM. Sulfur + molasses suppressed numbers of this pest only after the second application and bushes also showed signs of recovery by overgrowing bronzing symptoms. This was a rather surprising finding, given that Sulfur-CARB (brand name) is formulated as a soil amendment used to increase soil oxidation, adjust pH, and stimulate microorganism populations, which can also be applied over the foliage (Sulfur-CARB label). We wanted to evaluate this product, since it was brought to our attention by some growers using it in their plantings against the SRM. This was not confirmed in the survey, since only one grower included this product in their list of pesticides. Despite this finding, we strongly recommend only using miticides registered as effective against spider mites (Tetranychidae) or SRM specifically. Despite the popularity of sulfur (Cosavet) for suppressing some mite pests, it was not effective at suppressing SRM during our trials.

Well-performing miticides are now available for use against SRM in SHB, but these tools are not yet being used by most blueberry growers in Florida. Blueberry pest management programs need to be informed about these tools to improve SRM suppression, pesticide rotations, and avoid reliance on broad-spectrum insecticides/miticides. The diversity of plant protection products responded to by the growers demonstrated the potential to develop good rotation programs if more miticides options are included. Nevertheless, only two miticides (bifenthrin and fenpyroximate) were included as used in the blueberry plantings. It is highly recommended to diversify the miticide options to avoid the development of miticide resistance and to enhance the natural enemy populations that may be contributing to the suppression of SRM.

In response to the needs identified, we have published various educational materials available to growers between 2020 and 2022, including extension publications and presentations. These address basic information to identify SRMs, their injury to blueberry foliage, and miticide products that should be included in the insecticide/miticide rotation plan of growers to mitigate mite infestations. Additionally, the 2020 miticide trials shown in this study were based on the miticide trials started in 2019 [9], with the addition of more pest management products suggested by growers during the blueberry meetings to demonstrate their potential to suppress SRM populations.

This research is the first report of predatory mites naturally occurring in SHB in Florida. We believe that the lack of broad-spectrum insecticide applications before and during the trials allowed predatory mites to migrate to the SRM-infested bushes. We also observed predatory mites feeding on the SRM in field observations. The significant percentage of samples including predatory mites overlapped with the time when the lowest numbers of SRMs were recorded. Thus, it appeared that the naturally occurring predators may have contributed to the suppressive effects of some miticides. Our hypothesis is based on the life tables of SRM and the phytoseiid mite *Amblyseius herbicolis*, indicating an increased population growth for the predatory mites when feeding on SRM in laboratory conditions [23]. It is not surprising to record predatory mites attracted to the SRM populations in the blueberry bushes during our study since several natural enemies have been found in association with *O. ilicis* in other crops such as coffee plantings in Brazil [20]. Some of these were reported as phytoseiids from the *Amblyseius* genera, like the one species identified during our trials in Florida’s SHBs. Finally, further investigations are needed to further clarify the effects of naturally occurring predatory mites on SRM populations in SHB and their potential to be integrated with miticide and insecticide rotation programs.

## 5. Conclusions

The spread of invasive and secondary pests requires blueberry pest management programs to adapt. The current study identifies grower awareness of SRM as an emerging pest. Additionally, it demonstrated fenpyroximate and fenazaquin as the best-performing miticidal tools to effectively suppress SRM. These miticides were recently registered for use in SHB.

This research documented for the first time two species of phytoseiid mites associated with SRM populations in SHB plantings in Florida: *N. ilicis* and *Amblyseius* spp. Populations of these naturally occurring predators could be enhanced by reducing the applications of broad-spectrum products against key insect pests. Additionally, selective miticides could be used to suppress SRM outbreaks, while at the same time maintaining natural enemy populations, as shown in this study.

Finally, establishing action thresholds is vital to designing management programs for SRM, in addition to evaluating cultivar preferences or susceptibility to SRM infestations. Likewise, continuing educational programs highlighting these tools is vital to spread the word about these IPM tactics and further grower assessments are important to identify any changes in growers’ approaches in the future.

## Figures and Tables

**Table 1:** List of miticides and recommended rate tested for control of southern red mites, *O. ilicis*.

Treatment (Active Ingredient, AI)	Miticide (Brand Name)	Product Rate: AI/ha	Manufactory
Spiromesifen	ALPB2017	1.25-L	Bayer, St. Louis, MO, USA
Acequinocyl	Kanemite^®^ 15SC	2.07-L	Arysta LifeScience, LLC, Cary, NC, USA
Sulfur + molasses	Sulfur-CARB™	3% *v*/*v*	Terra Feed, LLC, Plant City, FL, USA
Sulfur	Cosavet^®^ DF	13.6-kg	Sulfur Mills LTD, Mumbai, India
Bifenazate	Acramite^®^ 4SC (low rate)	0.88-L	Arysta LifeScience, LLC, Cary, NC, USA
Bifenazate	Acramite^®^ 4SC (high rate)	1.18-L	Arysta LifeScience, LLC, Cary, NC, USA
Fenpyroximate	Portal^®^ EC	2.38-L	Nichino America, Inc., Wilmington, DE, USA
Fenazaquin	Magister^®^ SC	2.65-L	Gowman Co., Yuma, AZ, USA
Control (water)	NA	NA	NA

NA: not applicable.

**Table 2:** Number of growers (n = 37) using each of the 26 cultivars reported in the survey.

Variety	No. of Growers
Abundance	1
Arcadia	12
Avanti	5
Chickadee	6
Emerald	21
Endura	3
Farthing	7
Flicker	3
Indigocrips	1
Jewel	17
Jolies	1
Kestrel	8
Kirra	2
Meadowlark	15
Myra	1
Optimus	5
Primadonna	5
Rebel	1
San Joaquin	1
Scintilla	3
Snowchaser	2
Springhigh	7
Star	1
Sweetcrisp	2
Ventura	2
Winter Bell	5

**Table 3:** Number of growers (n = 37) using each of the 20 insecticides or miticides reported in the survey.

Brand Name	Active Ingredient (AI)	Mode of Action (MoA)	No. of Growers
Admire	Imidacloprid	4A	2
Assail	Acetamiprid	4A	4
Apta	Tolfenpyrad	21A	5
Avio	Abamectin	6	1
Brigade	Bifenthrin	3A	2
Delegate	Spinetoram	5	4
Entrust	Spinosad	5	1
Exirel	Cyantraniliprole	28	1
Gylon	Chlorfenapyr	13	1
Malathion	Malathion	1B	3
Mustang	Zeta-cypermethrin	3A	3
Movento	Spirotetramat	23	2
Neem	Azadirachtin	UN	1
Oil	Oil	UNE	2
Portal	Fenpyroximate	21A	6
Pyganic	Pyrethrins	3A	1
Sulfur	Sulfur	UN	2
Sulfur-CARB	Sulfur + molasses	UN	1
Sultan	Cyflumetofen	25A	1
Venerate	Burkholderia spp.	UNB	1

## Data Availability

The data presented in this study are available on request from the corresponding author. The data are not publicly available due to privacy restrictions.
